# PtCo-excavated rhombic dodecahedral nanocrystals for efficient electrocatalysis†

**DOI:** 10.1039/d0na00717j

**Published:** 2020-08-29

**Authors:** Cong Shen, Xuemin Li, Yajing Wei, Zhenming Cao, Huiqi Li, Yaqi Jiang, Zhaoxiong Xie

**Affiliations:** State Key Laboratory of Physical Chemistry of Solid Surfaces & Department of Chemistry, College of Chemistry and Chemical Engineering, Xiamen University Xiamen 361005 P. R. China yqjiang@xmu.edu.cn zzxie@xmu.edu.cn +86-592-2183360

## Abstract

Platinum (Pt)-based catalysts have shown excellent catalytic performance in many fields, particularly for the oxygen reduction reaction (ORR) and direct oxidation of small fuel molecules. Further development of Pt-based electrocatalysts relies on the morphology design of Pt-based alloy nanocrystals (NCs) with highly accessible and active surface sites to optimize Pt atomic utilization. In this work, we reported PtCo-excavated rhombic dodecahedral (ERD) NCs consisting of the self-assembly of 24 ultrathin nanosheets synthesized by a simple wet chemical method. The morphology can be regulated from convex to excavated polyhedra by controlling the amount of formaldehyde and the molar ratio of the Co/Pt precursor. The as-prepared PtCo ERD NCs/C catalyst exhibits excellent ORR performance, which has about 12 times higher specific activity and 6 times higher mass activity than the commercial Pt/C catalyst. It also displays good electrocatalytic ability towards methanol oxidation, in which the specific activity and mass activity are about 6 times higher and 2 times higher than the commercial Pt/C, respectively. Their enhanced activity is attributed to the excavated structure and alloy feature.

## Abstract 2

We successfully synthesized excavated rhombic dodecahedral PtCo NCs using a wet chemical method. This structure and the alloy nature render the PtCo ERD NCs a large electrochemically active surface area and an excellent catalyst for OER and the methanol oxidation reaction.

## Graphical abstract

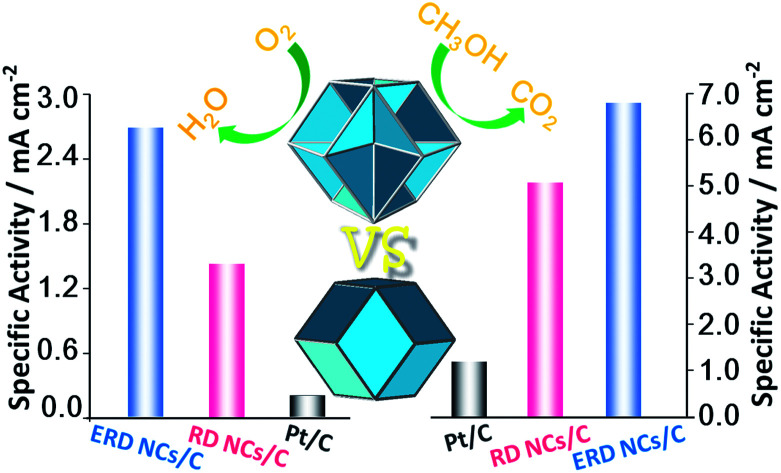

## 1. Introduction

Pt-Based nanocrystals (NCs) are the most promising catalysts for wide applications in oil refinement, fine chemicals, and fuel-cells. Considering the expensive price and limited availability of the noble-metal Pt, the controllable synthesis of noble metal nanocrystals with well-defined size, morphology, specific surface structure and composition is essential to maximize its atomic utilization efficiency and realize superior catalytic performance.^1–5^ Recent advances reveal that well-faceted noble metal NCs with concave morphology have been emerging as more attractive electrocatalyst candidates than their convex counterparts for the conversion of small organic molecules by oxidation/oxygen reduction in fuel cells.^6–13^ The excavated structure is the extreme example of deep concave morphology, which is constructed from the self-assembly of ultrathin nanosheets with merits of large surface area/volume ratios, specific exposed facets and less aggregation. Owing to their unique structure and abundant active sites on the surface, the noble metal-based NCs with excavated structure exhibit enhanced performance in electrocatalysis.^14–17^ Besides the optimization of the morphology and surface structure, the partial replacement of Pt with less-expensive transition metals, such as Co, Ni, Cu, Fe, Ti, and V, has proved to be an important strategy to enhance the catalytic performance of Pt-based NCs.^3–5,18–22^ Among a series of binary PtM alloys, the PtCo alloy NCs have been recognized as one of the most promising electrocatalysts towards oxygen reduction reaction (ORR), which can significantly reduce the cost of fuel cells, while maintaining performance.^23–27^ Therefore, PtCo alloy NCs with excavated structure can be a kind of robust catalyst having outstanding performance with high activity and high durability.

However, according to the basic principles of thermodynamics, crystals tend to adopt a convex polyhedral shape with the minimum surface energy, namely Gibbs–Wulff polyhedra. The excavated morphology with negative surface curvature is thermodynamically unfavourable in crystal growth.^28,29^ Consequently, the majority of PtM alloy NCs obtained to date are convex polyhedra, and only a few concaved/excavated structures have been recently explored. Even though their synthetic methods were roughly classified into a thermodynamic or kinetic approach involved in the use of a capping reagent, selective chemical etching or seed-mediated overgrowth,^30–34^ the growth mechanism of the excavated structures is still not well understood and the rational synthesis is far beyond our knowledge.

In this work, we successfully synthesized excavated rhombic dodecahedral (ERD) PtCo NCs using a simple wet chemical method. Detailed analysis showed that the excavated structure was constructed from an orderly assembly of 24 ultrathin nanosheets. Both formaldehyde and a molar ratio of Co/Pt play vital roles in constructing this excavated morphology. This unique excavated structure and the alloy nature provide the as-prepared PtCo ERD NCs with a large electrochemically active surface area and an excellent catalytic property towards the oxygen reduction reaction and the methanol oxidation reaction (MOR).

## 2. Experimental section

### 2.1 Materials

Oleylamine (C_18_H_37_N) was purchased from J&K Scientific, Ltd. Oleic acid (C_18_H_34_O_2_, 90%) was purchased from Alfa Aesar. Chloroplatinic acid (H_2_PtCl_6_·6H_2_O), cobalt(ii) acetate tetrahydrate (Co(Ac)_2_·4H_2_O), formaldehyde (HCHO), ethanol (C_2_H_5_OH), hexane (C_6_H_14_) and *n*-butylamine (C_4_H_11_N) were purchased from Sinopharm Chemical Reagent Co., Ltd. All chemicals were of analytical reagent grade and directly used after purchase without further purification.

### 2.2 Synthesis of PtCo ERD NCs and PtCo RD NCs

In a typical synthesis of PtCo ERD NCs, 0.075 mmol of Co(Ac)_2_·4H_2_O and 0.025 mmol of H_2_PtCl_6_·6H_2_O were added to a beaker containing 6 mL of oleylamine and 2 mL of oleic acid under magnetic stirring. After the mixture changed color into a clear solution, 300 μL of formaldehyde solution was injected and the mixture was stirred for 30 min. Then, the mixture was transferred to a Teflon-lined stainless-steel autoclave with a capacity of 25 mL, and the sealed vessel was heated from room temperature to 220 °C in around 70 min. The sealed vessel was kept at this temperature for 10 h before it was allowed to cool to room temperature naturally. The product was collected by centrifugation (9000 rpm for 3 min), and washed several times with a mixture solution of ethanol and hexane (the volume ratio 7 : 3) to remove impurities. For the PtCo rhombic dodecahedral (RD) NCs, the synthesis procedure is almost the same, but there is no need for the formaldehyde solution.

Both as-synthesized PtCo NCs were deposited onto carbon supports (Vulcan XC-72) before the electrochemical tests. Typically, the supernatant of the as-synthesized PtCo NCs was discarded first, and then 5 mL of *n*-butylamine and 3 mg of XC-72 were added to the samples. The mixed solution was transferred to a bottle with a capacity of 20 mL, and sonicated in an ice-bath for 1 h. After it was magnetically stirred for 3 days (1500 rpm) at 25 °C, the PtCo NCs/C catalysts were collected by centrifugation and washed with ethanol several times.

### 2.3 Characterization of sample

The powder X-ray diffraction (XRD) patterns were recorded on a Rigaku Ultima IV X-ray diffractometer with Cu Kα radiation to study the crystallographic and compositional information of the samples. The morphology and crystal structure of the as-prepared products were observed by scanning electron microscopy (SEM, Hitachi S4800) and high-resolution transmission electron microscopy (HRTEM, JEM 2100) with an acceleration voltage of 200 kV. High-angle annular dark-field scanning transmission electron microscopy (HAADF-STEM) and energy-dispersive X-ray spectroscopy (EDS) were performed with a FEI TECNAI F30 microscope operated at 300 kV. All TEM samples were prepared by depositing a drop of the diluted suspension in ethanol onto a carbon film-coated molybdenum grid. The precise content of every element in the sample was determined by inductively coupled plasma atomic emission spectroscopy (ICP-AES, Baird PS-4).

### 2.4 Electrochemical measurements

All electrochemical measurements were recorded on an electrochemical workstation with three-electrode configuration (CHI 660E, Shanghai Chenhua, Shanghai). A glassy carbon rotating disk electrode (RDE, 5 mm in diameter, Pine Research Instrumentation) equipped with a catalyst was used as the working electrode, a carbon rod electrode served as the counter electrode, and a saturated calomel electrode (SCE) was used as the reference electrode at 25 °C. Before each experiment, the glassy carbon electrodes were carefully polished and cleaned. Then, the catalysts were transferred to their surfaces and dried at room temperature.

For the oxygen reduction reaction, the total platinum loading for each experiment was 2 μg. The cyclic voltammogram (CV) curve was scanned until it was stable in a N_2_-saturated HClO_4_ solution (0.1 M) with a scan rate of 100 mV s^−1^ from 0.05 to 1.1 V (*vs.* RHE). The ORR polarization curve was performed in an O_2_-saturated HClO_4_ solution (0.1 M) at a scan rate of 10 mV s^−1^ and a rotation rate of 1600 rpm. The specific and mass activities were studied at 0.9 V (*vs.* RHE), and depicted as kinetic current densities normalized to the real active surface area (*Q*/*q*_0_) and the loading Pt mass. For the methanol oxidation reaction, the total platinum loading for each experiment was 3 μg. The CV curve was scanned to a stable profile in a N_2_-saturated H_2_SO_4_ solution (0.5 M) at a scan rate of 50 mV s^−1^. Methanol oxidation was carried out in a N_2_-saturated solution of 0.5 M H_2_SO_4_ + 0.5 M CH_3_OH at a scan rate of 50 mV s^−1^. The ECSA of the catalyst was calculated by the following formula: ECSA = *Q*/(*q*_0_·*m*), where *Q* was determined by the area of the hydrogen desorption peaks in the CV measurement performed in the corresponding N_2_ saturated electrolyte, *q*_0_ is 210 μC cm^−2^, and *m* is the loading Pt mass.

## 3. Results and discussion

**Fig. 1:**
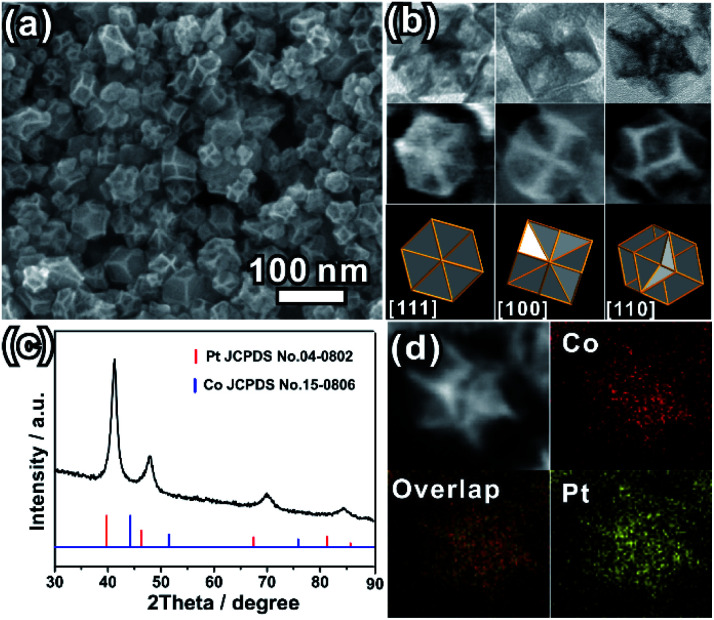
(a) SEM image of the PtCo ERD NCs; (b) high-magnification TEM images and SEM images of an individual PtCo ERD NC, and the corresponding schematic models viewed along the [111], [100] and [110] directions; (c) XRD pattern of the PtCo ERD NCs; (d) HAADF-STEM image and EDS maps of a single PtCo ERD NC.

The as-prepared PtCo ERD NCs were synthesized *via* wet-chemical co-reduction of Co(Ac)_2_·4H_2_O and H_2_PtCl_6_·6H_2_O in the presence of formaldehyde solution. The representative SEM image shown in Fig. 1a and the TEM image (Fig. S1a, ESI†) show that the as-prepared product is composed of concave NCs with an average size of 40.0 nm (size range from 26.0 nm to 58.0 nm, Fig. S1b, ESI†). The high-magnification TEM and SEM images (Fig. 1b) of a single NC oriented in three different directions show the distinct contrast distribution between the edges and faces, featuring a deeply concave structure. Fig. 1b illustrates the good match of the SEM/TEM images of an individual ERD NC with the schematic models of an excavated rhombic dodecahedron oriented in the [111], [100] and [110] directions. The results indicate that the shape of the as-prepared PtCo NCs is an excavated rhombic dodecahedron, and this excavated rhombic dodecahedron is constructed from 24 ultrathin nanosheets with 3.4 nm thickness (Fig. S2, ESI†). Referring to our previous work on PtCu_3_ ERD alloy NCs and the results of other relevant reports, the main surface of the nanosheets could be the {110} facet.^14,35,36^ The XRD pattern (Fig. 1c) can be indexed to a face-centred cubic (fcc) structure. The diffraction peaks of the fcc structure were located between those of the standard fcc Pt (JCPDS no. 04-0802) and fcc Co (JCPDS no. 15-0806), suggesting the successful alloying of the Pt and Co elements. The lattice parameter of the PtCo ERD NCs was calculated to be 3.786(7) Å. According to Vegard's law, which illustrates that the lattice parameter of an alloy is linearly related to its composition, the Co content was estimated to be 36 at% in the PtCo ERD NCs. The composition of PtCo ERD NCs was further tested by energy dispersive spectroscopy (Fig. S3, ESI†). 35 at% of the Co content is very close to the value obtained from the XRD pattern. As depicted by the HAADF-STEM image and the EDS mapping of a single PtCo ERD NC shown in Fig. 1d, and the line scanning data shown in Fig. S4 in the ESI,† the homogeneous spatial distribution of the Pt and Co elements throughout the entire nanocrystal further manifests the formation of the PtCo solid-solution alloy NCs. It can be concluded that the alloy PtCo NCs were successfully synthesized, and they adopt the unique excavated rhombic ultrathin nanosheets with {110} main facets.

As mentioned above, the formation of excavated structures is thermodynamically unfavoured. To better understand the formation process, a time-dependent experiment was conducted. The intermediates formed at different reaction times were investigated by TEM and XRD techniques. The TEM images (Fig. S5, ESI†) show that the excavated NCs were formed at the very beginning with an average size of 21.4 nm (Fig. S5, ESI†). After that, the excavated NCs grew large with increasing reaction time. The average size of the ERD NCs was increased to 32.6 nm at 1 h, 36.5 nm at 4 h, and finally 38.4 nm at 8 h. The size was almost equal to that of the final product at 10 h (Fig. 1a). It is noteworthy that the Pt and Co contents of the ERD NCs were maintained at different reaction times. The XRD patterns (Fig. S6, ESI†) show that each diffraction peak of the same diffraction index overlaps at the same two theta angles. These results imply that the excavated structure and the ultrathin nanosheets formed at the very beginning, and grew large with increasing reaction time. Different from selective chemical etching or erosion,^10,33^ the formation of the as-prepared PtCo ERD NCs in this case probably undergoes a surface capping process, such as the reported excavated cubic PtSn NCs formed in the presence of PVP, and the excavated octahedral PtCo formed with the selective capping of Br^−^ ions.^31,32^

**Fig. 2:**
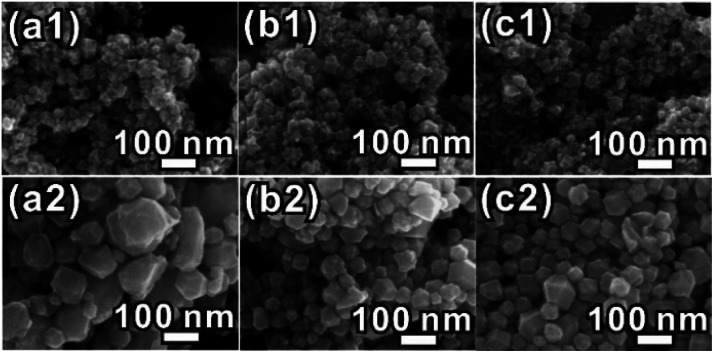
SEM images of the Pt–Co alloy NCs formed from different amines with or without formaldehyde, while keeping the other conditions the same. (a1 and a2) *n*-Butylamine; (b1 and b2) *n*-octylamine; (c1 and c2) octadecylamine.

To figure out the influence factor of the ERD morphology formed at the very beginning, we designed a series of control experiments. When oleylamine was replaced by *n*-butylamine, *n*-octylamine or octadecylamine in the presence of 300 μL of formaldehyde, the morphologies of the products are all concave or excavated, which is similar to the product obtained using oleylamine (Fig. 2(a1–c1)). However, the morphologies of the products were all convex polyhedra (Fig. 2(a2–c2)) when formaldehyde was not used in the synthesis system described above. All of the XRD patterns (Fig. S7, ESI†) confirmed that the products are PtCo alloy NCs. In contrast, when the formaldehyde volume was decreased to 100 μL, the obtained NCs were convex polyhedra as well (Fig. S8, ESI†). Upon increasing the dosage of the formaldehyde solution to more than 300 μL, such as 500 μL or 700 μL, the surfaces of the obtained NCs became very rough and the ERD structures collapsed (Fig. S8b and c, ESI†). The uniform and well-defined excavated NCs were produced when using only ∼300 μL of formaldehyde (Fig. 1a). We realized that the formaldehyde solution and the amount used have significant influence on the formation of the PtCo ERD NCs; namely, an appropriate amount of formaldehyde is feasible for constructing polyhedra with negative curvature and forming nanosheets as the building blocks. Few similar phenomena have been found in some related research studies. For example, Zheng and co-workers reported that the concavity of Pd tetrahedra was highly dependent on the amount of formaldehyde added.^6^ We also found that formaldehyde was a crucial factor in the formation of Rh ultrathin nanosheets in the previous work.^37^

**Fig. 3:**
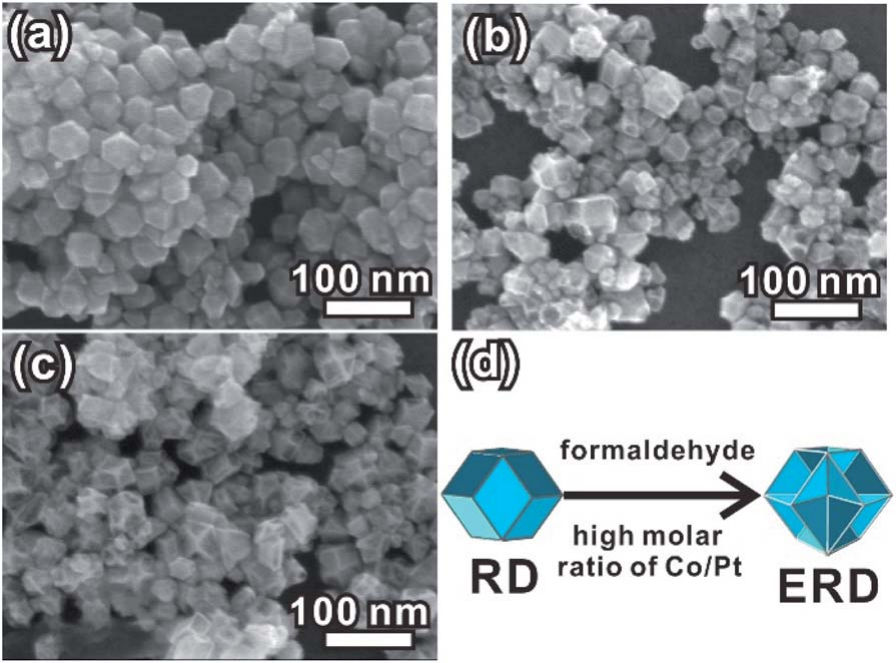
SEM images of the PtCo alloy NCs synthesized from different molar ratios of Co and Pt precursors with 300 μL of formaldehyde solution. (a) 1 : 1; (b) 2 : 1; (c) 5 : 1. (d) Schematic diagram for the morphology evolution from RD to ERD.

We further evaluated the other factor affecting the formation of the excavated structure. By changing the initial molar ratio of the Co and Pt precursors, a series of rhombic dodecahedral structures with different concavity degrees appeared (Fig. 3). The XRD patterns shown in Fig. S9 in the ESI† show that all of the formed products are PtCo alloy NCs. The SEM image (Fig. 3a) demonstrates that the convex rhombic dodecahedra formed at a Co/Pt molar ratio of 1 : 1, while a partial concave structure was formed when the molar ratio was tuned to 2 : 1 (Fig. 3b). When the concentration of cobalt acetate was further changed to a Co/Pt ratio of 5 : 1, the result was drastically different. The formed NCs became smaller, and part of them were structurally collapsed (Fig. S10, ESI†). Our experiments reveal that the perfect excavated rhombic dodecahedral morphology can be formed when the Co/Pt molar ratio is about 3 : 1 (Fig. 3c). In addition, when the Co/Pt ratio reached 5 : 1, we found an unexpected phenomenon in which the concave PtCo NCs can be formed even in the absent of formaldehyde (Fig. S11, ESI†). From a crystallographic view, a crystal tends to grow a convex polyhedral structure under equilibrium conditions. Therefore, the formation of an excavated structure is more likely to occur in a kinetically controlled process. This will hinder the formation of normal nuclei following the Gibbs–Wulff construction rule. The excessive amount of Co^2+^ ions accelerates the reduction of Co^2+^ ions, and is beneficial to co-reduction and the growth of PtCo alloy NCs due to its more negative reduction potential compared to that of the Pt^4+^ ions.^38^ We performed a contrast synthetic experiment with or without formaldehyde solution, while keeping the molar ratio of Co and Pt precursor at 3 : 1 and 1 h of reaction time. As shown in Fig. S12,† no product was obtained (left bottle) without formaldehyde. The solution color is the same as that of an unreacted solution of precursor Co(Ac)_2_·4H_2_O. Conversely, the PtCo alloy nanoparticle (black product) can be found in the bottom of the right bottle, in which 300 μL of formaldehyde was added. The results in our case suggest that speeding up the reduction rate of transition metal ions is beneficial to the formation of the concave or excavated structure, and the large Co/Pt initial ratio plays a valuable role.

For comparison, a detailed characterization was conducted for the PtCo alloy NCs obtained without formaldehyde. The large-scale SEM image (Fig. S13a, ESI†) and TEM image (Fig. S14, ESI†) show that the shape of the PtCo alloy NCs is a well-defined rhombic dodecahedral, and the element component calculated from XRD pattern (Fig. S13b, ESI†) is about Pt_64_Co_36_. This is very close to that of the as-prepared PtCo ERD NCs. The high-resolution TEM image (Fig. S13c, ESI†) of the single PtCo NC matches well with the schematic model of the rhombic dodecahedron oriented along the [011] direction (Fig. S13e, ESI†). Combining the above results, the morphology evolution of PtCo NCs from a convex RD to an excavated RD is schematically demonstrated in Fig. 3d. It is reasonable to believe that this morphology evolution is induced by the synergetic control of formaldehyde and the molar ratio of Co/Pt.

**Fig. 4:**
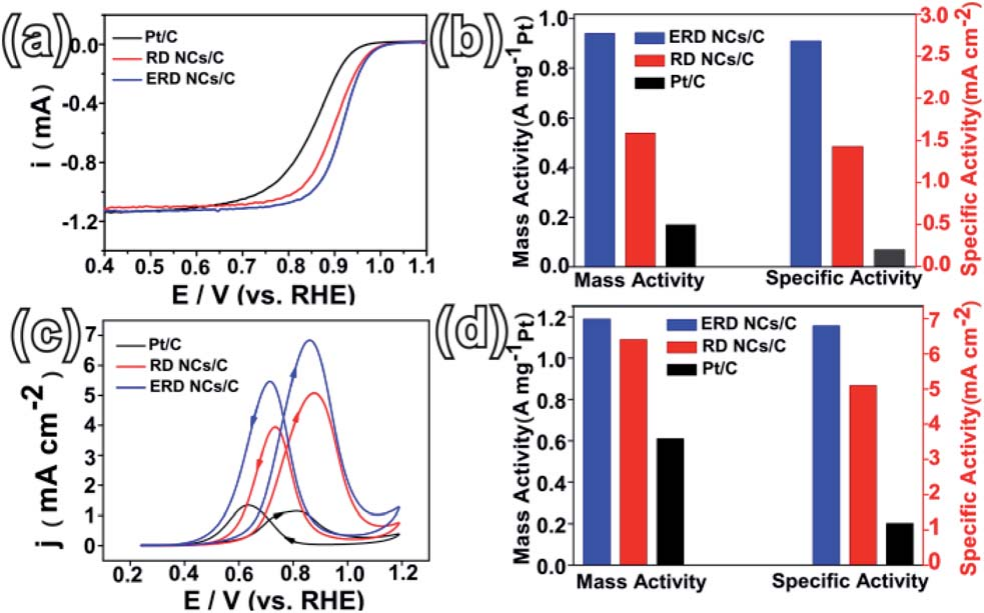
(a) ORR polarization curves of PtCo ERD NCs/C, PtCo RD NCs/C and commercial Pt/C; (b) histogram of the specific activity and mass activity of ORR at 0.9 V (*vs.* RHE) for the three catalysts; (c) CV curves of MOR; (d) histogram of the specific activity and mass activity of MOR at 0.9 V (*vs.* RHE) for the three catalysts.

To evaluate the shape-dependent catalytic properties, ORR (in the cathode of PEMFCs) was studied using three catalysts, PtCo ERD NCs/C, PtCo RD NCs/C and commercial Pt/C. Both PtCo ERD and RD NCs were dispersed on Vulcan XC-72 carbon before measurement. All measurements were performed by making use of the RDE technique, and the loading of Pt on the three catalysts was consistent. The CV curves of different catalysts tested in N_2_-saturated 0.1 M HClO_4_ (Fig. S15a, ESI†) show obvious peaks associated with the hydrogen adsorption/desorption process in the potential range of 0.05–0.35 V, and the ECSA of the as-prepared PtCo ERD NCs/C was estimated to be 35.0 m^2^ g_Pt_^−1^. The polarization curves (Fig. 4a) show that the PtCo ERD and PtCo RD catalysts have more positive onset potentials than Pt/C, revealing that the adsorption of the oxygenate species on the surface of the PtCo NCs/C catalysts was much weaker and thus more active than Pt/C.^39^ The kinetic currents at 0.9 V *versus* RHE were normalized to the real active surface area and the mass loading of Pt to obtain the specific and mass activities, respectively. Fig. 4b demonstrates that the specific activities are 2.68, 1.43 and 0.2 mA cm^−2^ for PtCo ERD NCs/C, PtCo RD NCs/C and commercial Pt/C, respectively, and the mass activities are 0.94, 0.54 and 0.17 A mg_Pt_^−1^ for PtCo ERD NCs/C, PtCo RD NCs/C and Pt/C, respectively. Among the three catalysts, the as-prepared PtCo ERD NCs/C catalyst exhibits superior ORR performance over the commonly used commercial Pt/C in terms of both specific activity (∼12 times higher) and mass activity (∼6 times higher). Notably, we found that the activity of the as-prepared PtCo ERD NCs/C catalyst is comparable to the highest known reported values so far for Pt–Co catalysts, especially in terms of the specific activity (Table S1, ESI†). We also applied the catalysts to the methanol oxidation reaction, in which the MOR measurements were carried out in a mixture solution of 0.5 M H_2_SO_4_ and 0.5 M CH_3_OH. Fig. 4c shows that two anodic peaks were clearly observed during the forward and backward sweeps, and PtCo ERD NCs/C showed the highest activity among the three catalysts. It was found that the specific activity of 6.8 mA cm^−2^ and the mass activity of 1.2 A mg_Pt_^−1^ were about 6 times and 2 times higher than that of commercial Pt/C, respectively (Fig. 4d). The two PtCo NCs/C catalysts exhibited better performance than Pt/C, suggesting the enhanced activity of the as-prepared PtCo ERD NCs/C can be partially contributed to the alloy feature of the Pt and Co elements. It was demonstrated that the improved catalytic performance of the PtM alloys is due to the surface electronic structure modification (d-band centre) from the bimetallic synergetic effect, surface atomic arrangement improvement (bond distance and coordination number) and the possible lattice strain effect of Pt.^40–43^ On the other hand, it may also be ascribed to the unique excavated structure integrating the merits of the large surface area, which results in high atomic utilization efficiency and the feasible accessibility of the active sites. In addition, by careful observation of the TEM images, we found that the PtCo ERD catalyst is structurally stable due to less agglomeration, and the excavated structure can be maintained after electrochemical measurements (Fig. S16 and S17, ESI†). The stability of the as-prepared PtCo ERD NCs/C catalyst was evaluated by chronoamperometry carried out in a mixed solution of 0.5 M H_2_SO_4_ + 0.5 M CH_3_OH at 0.4 V (*vs.* SCE). The *i*–*t* curves (Fig. S18 ESI†) reveal a slow attenuation in the PtCo ERD NCs/C catalyst, and show a current density that is higher (by ∼1.5 times) than that of Pt/C after 1200 s of measurement.

## 4. Conclusion

In summary, PtCo excavated rhombic dodecahedral NCs consisting of an orderly assembly of 24 nanosheets were successfully synthesized *via* a facile wet-chemical reaction. The presence of formaldehyde and an excessive amount of initial Co precursor were found to be two vital factors in the formation of this unique excavated structure. They can tune the morphology evolution of the PtCo NCs from convex to excavated. The as-prepared PtCo ERD NCs possessing a large electrochemical active surface area are structurally stable. Benefitting from the excavated structure and the alloying feature, the catalyst PtCo ERD NCs/C manifests the best electrocatalytic performance on ORR and MOR in comparison to PtCo RD NCs and commercial Pt/C. In particular, PtCo ERD NCs/C exhibits 12 times and 6 times enhancement in specific activity and mass activity towards ORR against a commercial Pt/C, respectively. Ultimately, our development for the morphology-controlled synthesis may open a possibility of engineering other alloy NCs integrating the merits of an excavated architecture, large surface area and specific crystal facet for promising catalytic performance.

## Conflicts of interest

There are no conflicts to declare.

## Supplementary Material

NA-002-D0NA00717J-s001
