# Supplementary material for: PtCo-excavated rhombic dodecahedral nanocrystals for efficient electrocatalysis
Source: Nanoscale Adv. 2020 Aug 29;2(10):4881–6. doi: 10.1039/d0na00717j (PMC9418751; doi:10.1039/d0na00717j)
Supplement: NA-002-D0NA00717J-s001 [file NA-002-D0NA00717J-s001.pdf]

Electronic Supplementary Material (ESI) for Nanoscale Advances.
This journal is © The Royal Society of Chemistry 2020

Supplementary Information

PtCo excavated rhombic dodecahedral nanocrystals for efficient electrocatalysis

Cong Shen,[a] Xuemin Li,[a] Yajing Wei,[a] Zhenming Cao,[a] Huiqi Li,[a] Yaqi Jiang [a,*] and Zhaoxiong Xie [a,*]

[a.] State Key Laboratory of Physical Chemistry of Solid Surfaces & Department of Chemistry, College of Chemistry and Chemical Engineering, Xiamen University, Xiamen 361005 (P. R. China). E-mail: yqjiang@xmu.edu.cn; zzxie@xmu.edu.cn; Fax: (+) 86-592-2183047.

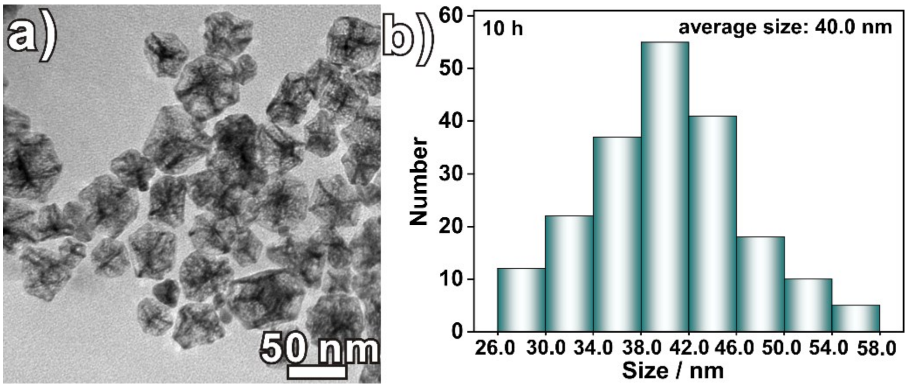

Fig. S1 (a) TEM image and (b) size distribution of the as-prepared PtCo ERD NCs based on statistics of 200 particles.

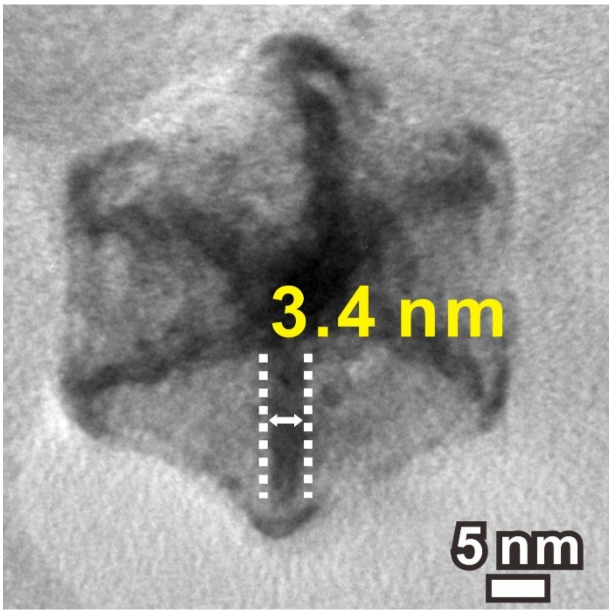

Fig. S2 High-magnification TEM image of a single PtCo ERD NC illustrating the thickness of a nanosheet.

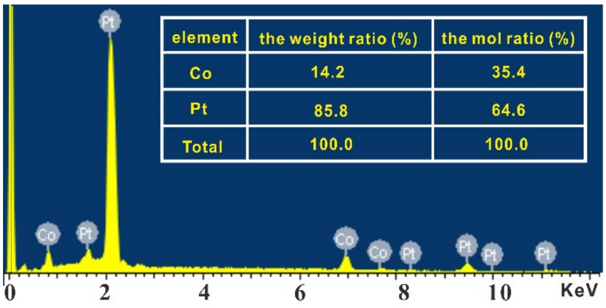

element	the weight ratio (%)	the mol ratio (%)
Co	14.2	35.4
Pt	85.8	64.6
Total	100.0	100.0

Fig. S3 EDS spectrum of the as-prepared PtCo ERD NCs.

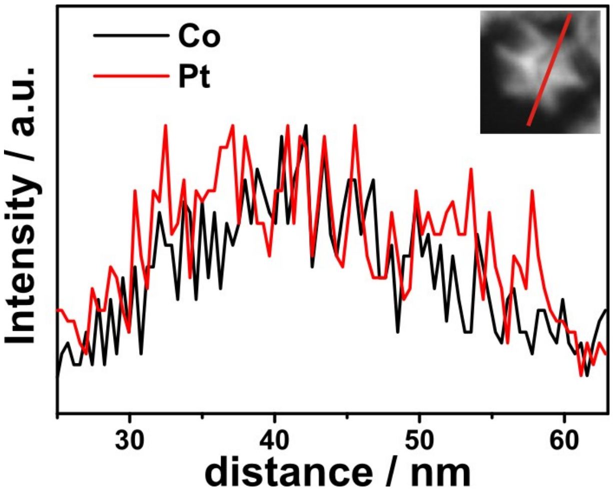

Fig. S4 Cross-sectional compositional line profiles of Pt and Co recorded across an individual PtCo ERD NC. The inset presents corresponding HAADF-STEM image.

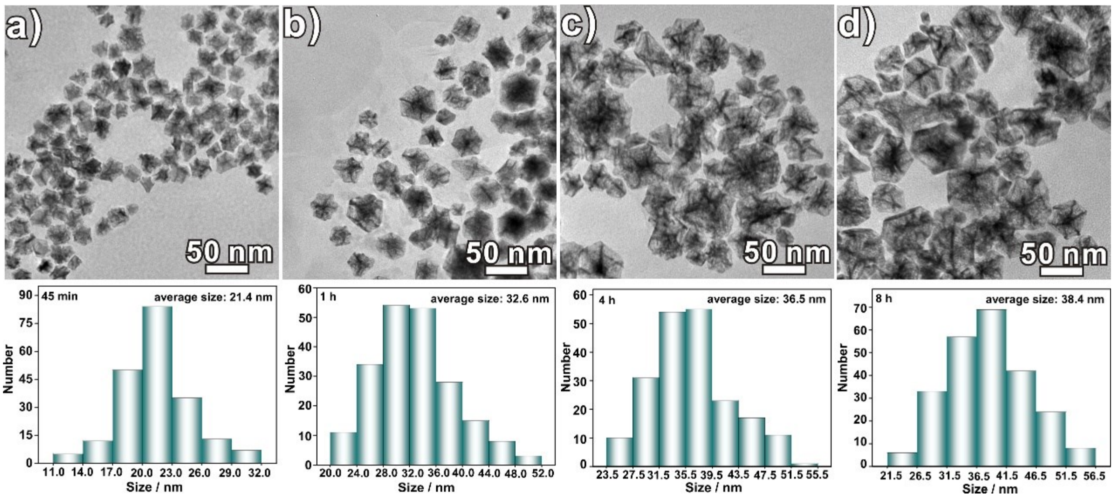

Fig. S5 TEM images and size distributions of the as-prepared PtCo ERD NCs obtained at different reaction time while keeping other conditions the same. (a) 45 min; (b) 1 h; (c) 4 h; (d) 8 h. The counted numbers of particles are 206, 206, 202, and 239, respectively.

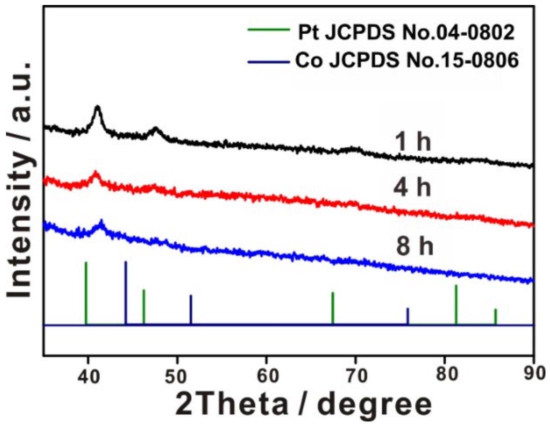

Fig. S6 XRD patterns of the as-prepared PtCo ERD NCs formed at different reaction times.

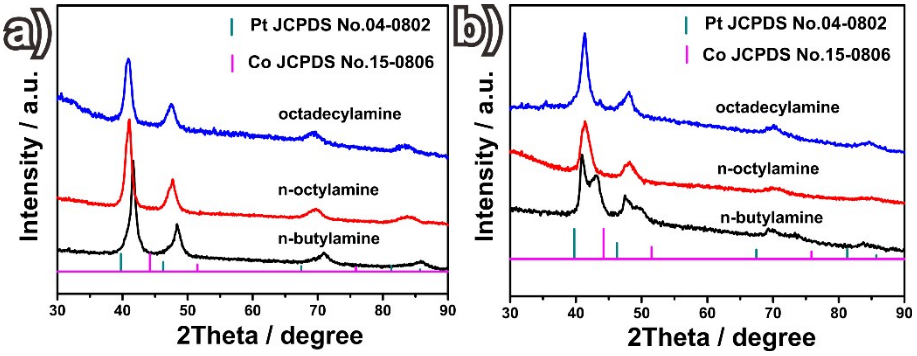

Fig. S7 XRD patterns of PtCo alloy NCs formed from different kinds of amines (a) with 300 μL of formaldehyde solution and (b) without formaldehyde solution while keeping other conditions the same.

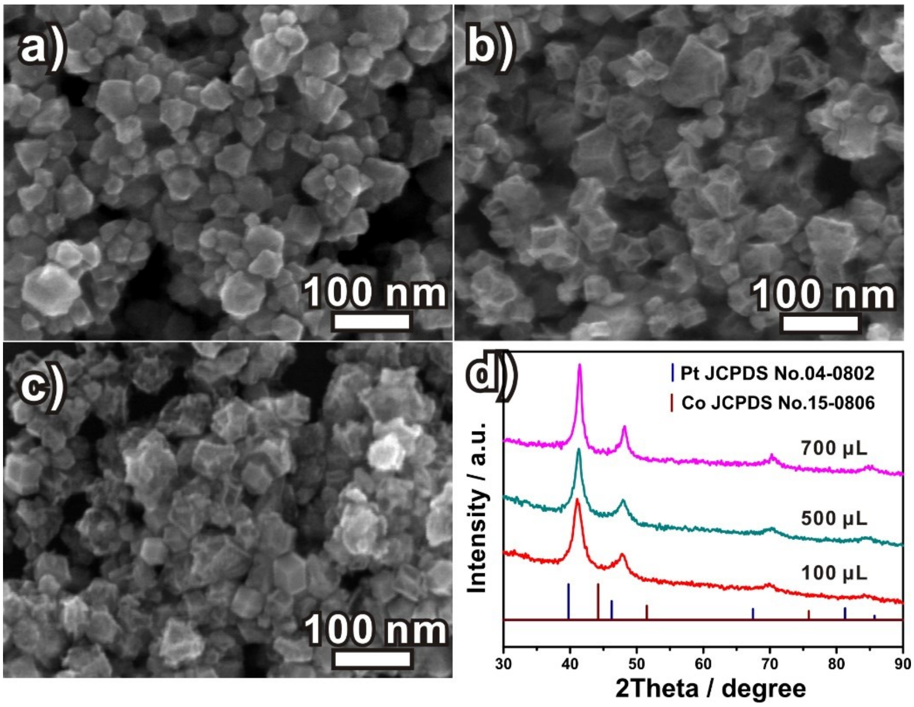

Fig. S8 SEM images of the PtCo NCs synthesized with different volume of formaldehyde while keeping other conditions the same. (a) 100 μL; (b) 500 μL; (c) 700 μL. (d) XRD patterns.

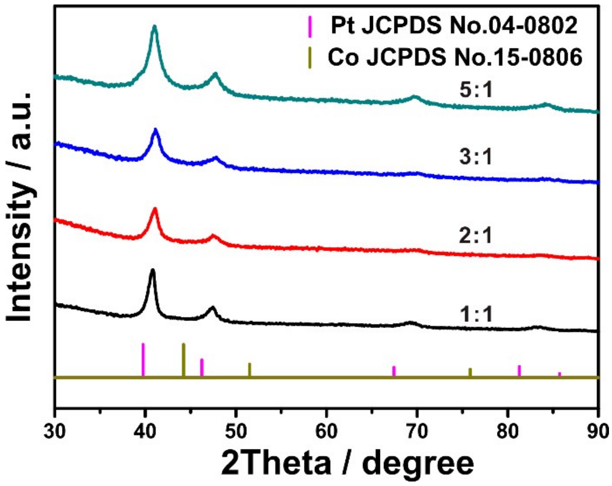

Fig. S9 XRD patterns of the PtCo alloy NCs synthesized from different molar ratios of Co and Pt precursors in the presence of 300 μL of formaldehyde.

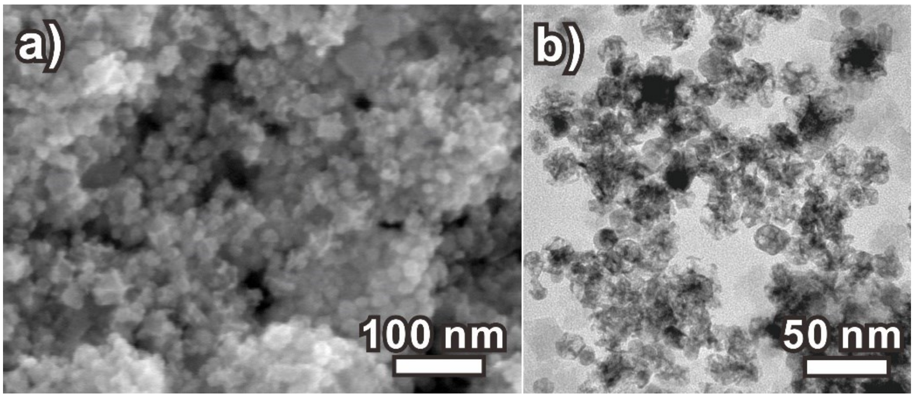

Fig. S10 (a) SEM image and (b) TEM image of the Pt-Co alloy NCs synthesized from Co/Pt molar ratio at 5:1 in the presence of 300 μL of formaldehyde while keeping other conditions the same.

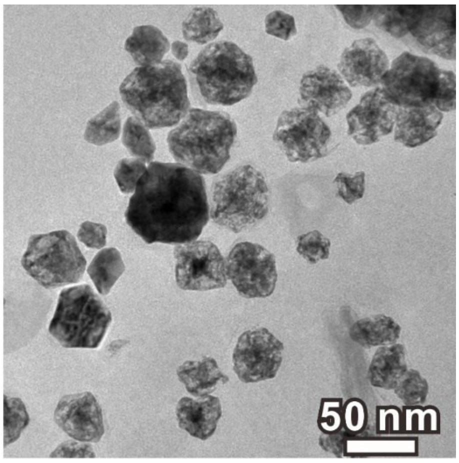

Figure S11 TEM image of product formed from the molar ratio of $Co(Ac)_2 \cdot 4H_2O$ and $H_2PtCl_6 \cdot 6H_2O$ at 5:1 in the absent of formaldehyde.

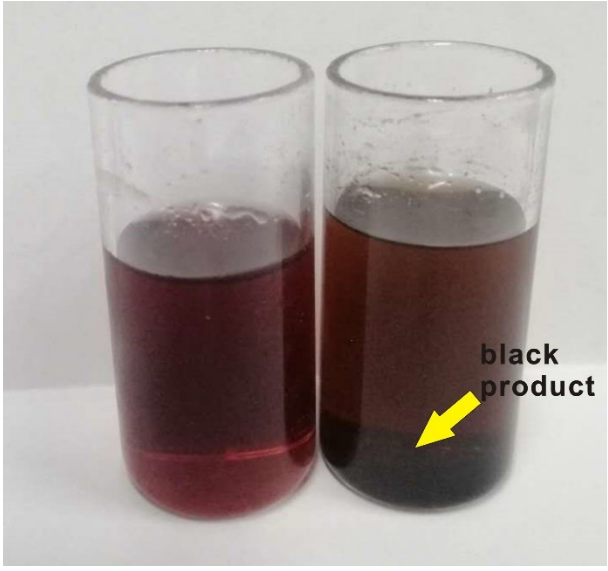

Fig. S12 The photography of the reactant solution obtained from the contrast synthetic experiment of 1 h of reaction, (left) without formaldehyde and (right) with 300 μL of formaldehyde solution.

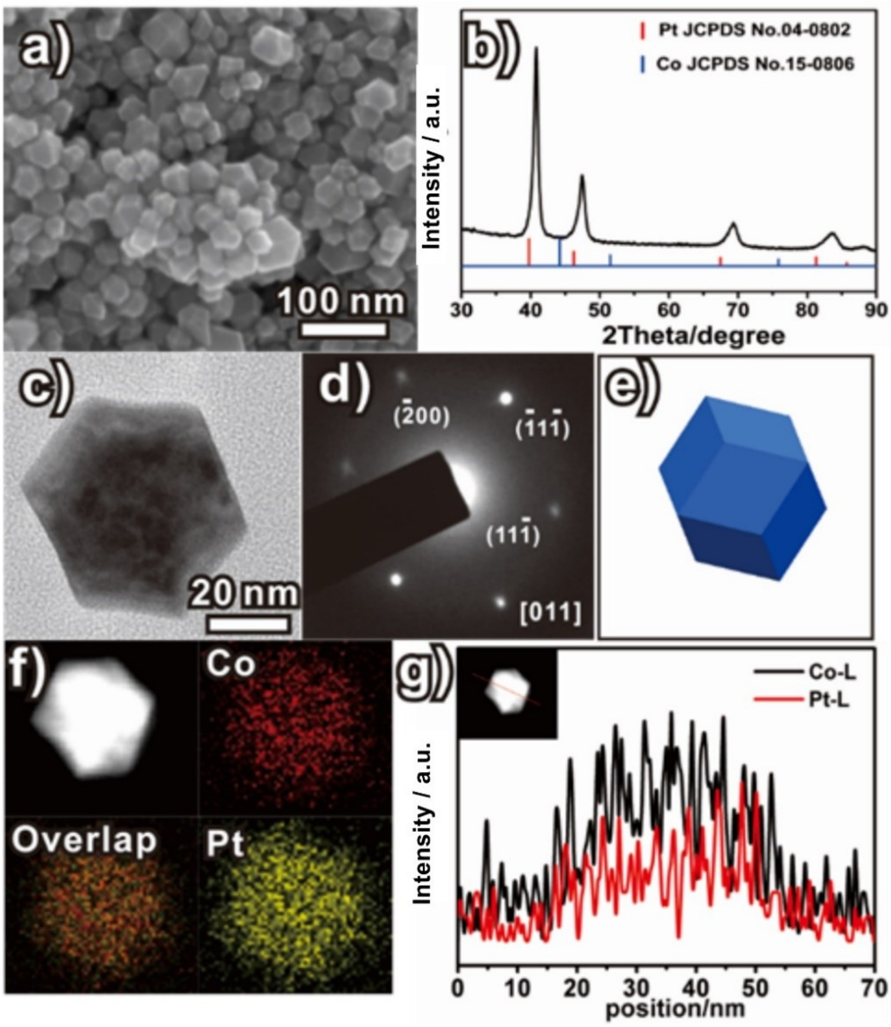

Fig. S13 (a) SEM image of PtCo RD NCs on a large scale; (b) XRD pattern of PtCo RD NCs; (c) TEM image and (d) the corresponding SAED image of an individual PtCo RD NC. (e) The schematic model of RD viewed along [011] direction. (f) HAADF-STEM image and HAADF-STEM-EDS maps of a single PtCo RD NC; (g) Cross-sectional compositional line profile of Pt and Co recorded across a single PtCo RD NC.

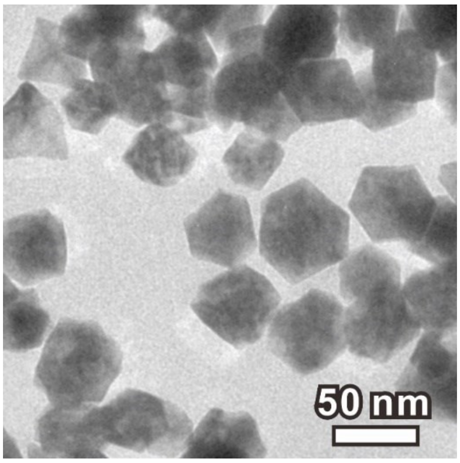

Fig. S14 TEM image of PtCo RD NCs on a large scale which obtained from molar ratio of $Co(Ac)_2 \cdot 4H_2O$ and $H_2PtCl_6 \cdot 6H_2O$ at 3:1 in the absent of formaldehyde.

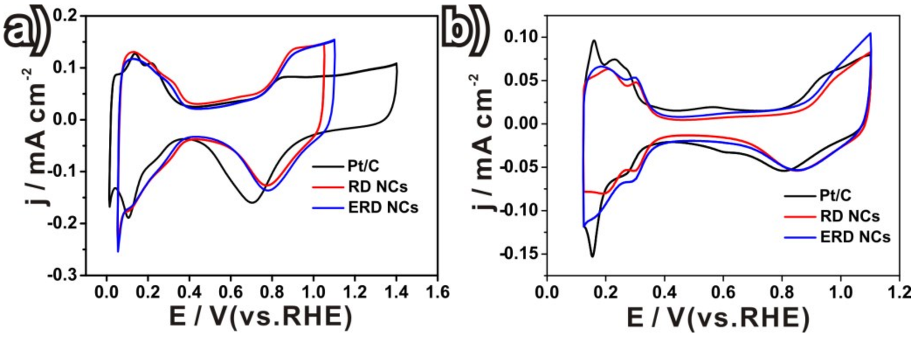

Fig. S15 (a) Cyclic voltammograms of as-prepared PtCo ERD NCs/C, RD NCs/C and Pt/C recorded in N_2-saturated $HClO_4$ solution (0.1 M) at scan rate of 100 mV s^{-1} and a rotation rate of 1600 rpm; (b) CVs recorded in N_2-saturated H_2SO_4 solution (0.5 M) at a scan rate of 50 mV s^{-1}.

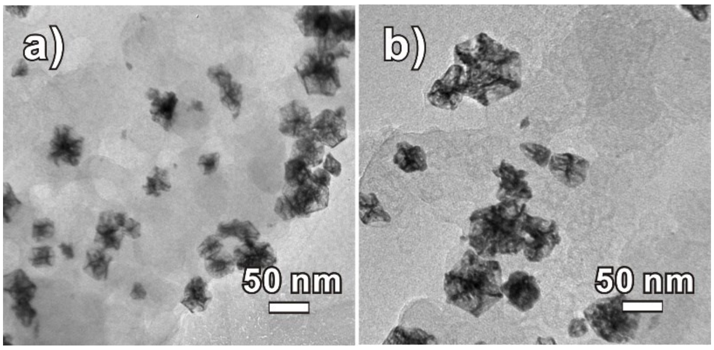

Fig. S16 (a-b) TEM images of PtCo ERD NCs/C catalyst after oxygen oxidation reaction.

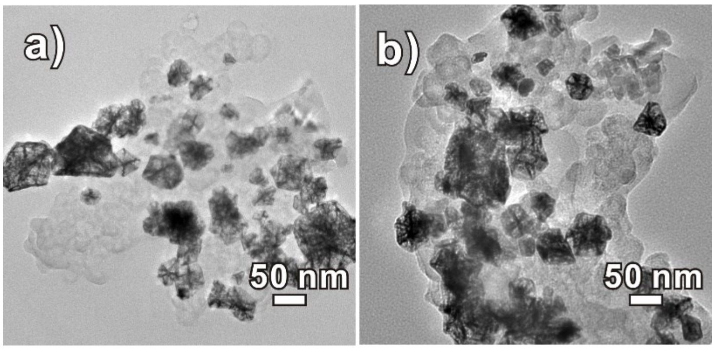

Fig. S17 (a-b) TEM images of PtCo ERD NCs/C catalyst after methanol oxidation reaction.

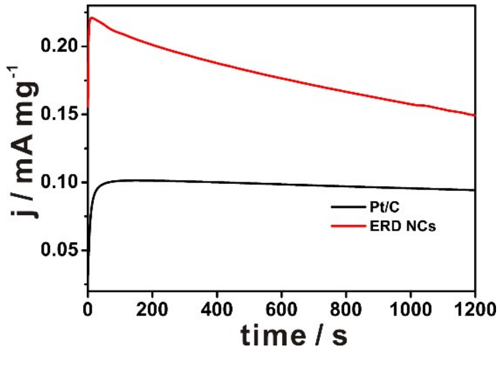

Fig. S18 Chronoamperometric measurements (i-t curves) of PtCo ERD NCs/C and commercial Pt/C in 0.5 M H_2SO_4 + 0.5 M CH_3OH at 0.4 V (vs. SCE).

Table S1 A comparison of the specific activities (SA) and mass activities (MA) of Pt-Co catalysts from literatures and this work.

Number	catalysts	electrolyte	SA@0.9V mA/cm^2	MA@0.9V A/mg$_{Pt}$	Ref.
1	PtCo@CNTs-MOF	0.1 M $HClO_4$	1.38	0.852	[1]
2	Pt-Co/C-PANI-500 °C	0.1 M $HClO_4$	1.29	1.33	[2]
3	PtCo/G-600	0.1 M $HClO_4$	0.524	0.952	[3]
4	Excavated PtCo octahedron	0.1 M $HClO_4$	1.53	0.35	[4]
5	Pt–Co NWs/C	0.1 M $HClO_4$		0.2914	[5]
6	Pt-Co NWs	0.1 M $HClO_4$	0.3564	0.179	[6]
7	leached PtCo/C_S#2	0.1 M $HClO_4$		0.313	[7]
8	PtCo–Pt (chem)	0.1 M $HClO_4$		0.38329	[8]
9	Ordered $PtCo_3$ H600	0.1 M $HClO_4$		0.72	[9]
10	$PtCo_3$/C	0.1 M $HClO_4$	~2.25	~1.13	[10]
11	Pt-skin Pt_3Co z-NWs/C	0.1 M $HClO_4$	5.6	2.2	[11]
12	Pt_3Co/C-$N_2H_4 \cdot H_2O$	0.1 M $HClO_4$	1.5	0.21	[12]
13	PtCo R-NW/C	0.1 M $HClO_4$	~1.04	~0.98	[13]
14	Pt/40Co-NC-900	0.1 M $HClO_4$	1.15	~0.24	[14]
15	H-PtCo@Pt_1N-C	0.1 M $HClO_4$	2.39	1.2	[15]
16	PtCo@HGS	0.1 M $HClO_4$	0.92 ±0.16	0.97 ± 0.19	[16]
17	Concave cubic Pt-Co	0.1 M $HClO_4$	0.439	0.237	[17]
18	PtCo@NC-10	0.1 M $HClO_4$	1.32	0.82	[18]
19	Pt 75 Co 25 /C(500)	0.1 M $HClO_4$	2.06	0.97	[19]
20	Pt-Co/C-PNIPAM	0.1 M $HClO_4$	1.45		[20]
21	PtCo RD NCs/C	0.1 M $HClO_4$	1.43	0.54	this paper
22	PtCo ERD NCs/C	0.1 M $HClO_4$	2.68	0.94	this paper

References

1. J. Wang, G. Wu, W. Wang, W. Xuan, J. Jiang, J. Wang, L. Li, W. F. Lin, W. Ding and Z. Wei, *Journal of Materials Chemistry A*., 2019, **7**, 19786-19792.
2. Y. Liu, N. Chen, F. Wang, Y. Cai and H. Zhu, *New Journal of Chemistry*., 2017, **41**, 6585-6592.
3. C. He, S. Zhang, J. Tao and P. K. Shen, One-step solid state synthesis of PtCo nanocubes/graphene nanocomposites as advanced oxygen reduction reaction electrocatalysts. *Journal of Catalysis*., 2018, **362**, 85-93.
4. Q. Chen, Z. Cao, G. Du, Q. Kuang, J. Huang, Z. Xie and L, *Nano Energy*., 2017, **39**, 582-589.
5. Liu, Z.; Yin, Y.; Yang, D.; Zhang, C.; Ming, P.; Li, B.; Yang, S. Efficient synthesis of Pt-Co nanowires as cathode catalysts for proton exchange membrane fuel cells. RSC Advances. 2020, 10, 6287-6296.
6. H. Lv, J. Wang, Z. Yan, B. Li, D. Yang and C. Zhang, *Fuel Cells*., 2017, **17**, 635-642.
7. Y. J. Wang, N. Zhao, B. Fang, H. Li, X. T. Bi, *RSC advances*., 2016, **6**, 34484-34491.
8. Y. S. Kang, D. Choi, H. Y. Park, S. J. Yoo, *Journal of Industrial and Engineering Chemistry*., 2019, **78**, 448-454.
9. Z. Wang, X. Yao, Y. Kang, L. Miao, D. Xia and L. Gan, *Advanced Functional Materials*., 2019, **29**, 1902987.
10. J. D. Lee, D. Jishkariani, Y. Zhao, S. Najmr, D. Rosen, J. M. Kikkawa, E. A. Stach and C. B. Murray, *ACS applied materials & interfaces*., 2019, **11**, 26789-26797.
11. M. Luo, Y. Sun, Y. Qin, S. Chen, Y. Li, C. Li, Y. Yang, D. Wu, N. Xu, Y. Xing, L. Wang, P. Gao and S. Guo, *Chemistry of Materials*., 2018, **30**, 6660-6667.
12. J. Huang, C. Ding, Y. Yang, G. Liu and W. B. Cai, *Chinese Journal of Catalysis*., 2019, **40**, 1895-1903.
13. X. Fan, S. Luo, X. Zhao, X. Wu, Z. Luo, M. Tang, W. Chen, X. Song and Z. Quan, *Nano Research*., 2019, **12**, 1721-1726.
14. X. X. Wang, S. Hwang, Y. T. Pan, K. Chen, Y. He, S. Karakalos, H. Zhang, J. S. Spendelow, D. Su and G. Wu, *Nano letters*., 2018, **18**, 4163-4171.
15. W. H. Lai, B. W. Zhang, Z. Hu, X. M. Qu, Y. X. Jiang, Y. X. Wang, J. Z. Wang, H. K. Liu and S. L.

Chou, *Advanced Functional Materials*., 2019, **29**, 1807340.

16. E. Pizzutilo, J. Knossalla, S. Geiger, J. P. Grote, G. Polymeros, B. Baldizzone, S. Mezzavilla, M. Ledendecker, A. Mingers and S. Cherevko, *Advanced Energy Materials*., 2017, **7**, 1700835.
17. Y. Ma, L. Yin, T. Yang, Q. Huang, M. He, H. Zhao, D. Zhang, M. Wang and Z. Tong, *ACS Appl Mater Interfaces*., 2017, **9**, 36164-36172.
18. N. Du, C. Wang, R. Long and Y. Xiong, *Nano Research*., 2017, **10**, 3228-3237.
19. D. S. Choi, A. W. Robertson, J. H. Warner, S. O. Kim and H. Kim, *Advanced materials*., 2016, **28**, 7115-22.
20. N. Jung, S. Bhattacharjee, S. Gautam, H. Y. Park, J. Ryu, Y. H. Chung, S. Y. Lee, I. Jang, J. H. Jang, S. H. Park, *NPG Asia Materials*., 2016, **8**, e237.
